# Machine vision-based detection of browning maturity in shiitake cultivation sticks

**DOI:** 10.3389/fpls.2025.1676977

**Published:** 2025-11-07

**Authors:** Zeting Liu, Jiuxiao Zhao, Wengang Zheng, Qiuxiao Song, Xin Zhang, Wei Liu, Feifei Shan, Ruixue Xu, Zuolin Li, Jing Dong, Pengfei Zhao, Yajun Wang, Mingfei Wang

**Affiliations:** 1 School of Mechanical Engineering and Automation, Dalian Polytechnic University, Dalian, China; 2 Intelligent Equipment Technology Research Center, Beijing Academy of Agriculture and Forestry Sciences, Beijing, China; 3 NongXin Science & Technology Co., Ltd., Beijing, China; 4 Shandong Qihe Biotechnology Co., Ltd., Zibo, Shandong, China; 5 Information Technology Research Center, Beijing Academy of Agriculture and Forestry Sciences, Beijing, China

**Keywords:** shiitake cultivation sticks, browning detection, image segmentation, YOLOv11, ResNet

## Abstract

**Introduction:**

Accurate monitoring of pigmentation changes during the browning stage of shiitake cultivation sticks is essential for assessing substrate maturity, forecasting mushroom emergence, and improving cultivation quality. However, current commercial detection methods lack objective, real-time, and quantifiable evaluation indicators for assessing the browning degree.

**Methods:**

This study proposes a two-stage image segmentation approach to address this issue. First, a novel VG-Stick-YOLOv11 model, built upon YOLOv11n-seg with VanillaNetBlock and GhostConv, was developed for real-time contour extraction and browning stage classification of shiitake sticks. Based on the extracted features, machine learning techniques facilitated rapid, semi-automatic annotation of browning regions, thereby constructing a segmentation dataset. Finally, the ResNet-Stick-UNet (RS-UNet) model was designed for precise browning region segmentation and area ratio calculation. The encoder utilizes ResNet50 with multi-branch inputs and stacked small kernels to enhance feature extraction, while the decoder incorporates a hybrid structure of grouped and depthwise separable convolutions for efficient channel fusion and detail preservation. A spatial attention mechanism was embedded in skip connections to emphasize large-scale browning regions.

**Results:**

The proposed VG-Stick-YOLOv11 achieved the best mIoU of 95.80% for stick contour extraction while markedly reducing parameters and computation. For browning region segmentation, RS-UNet achieved a high segmentation accuracy of 94.35% and an IoU of 88.56%, outperforming comparison models such as Deeplabv3+ and Swin-UNet. Furthermore, RS-UNet reduced the number of parameters by 36.31% compared to the ResNet50-U-Net baseline.

**Conclusion:**

The collaborative two-stage model provides an effective and quantitative solution for maturity detection of shiitake cultivation sticks during the browning stage. This work promotes the intelligent and standardized development of shiitake substrate cultivation.

## 1 Introduction

In recent years, the “domestic stick production and overseas fruiting” model has gradually matured, while the scale of China’s shiitake mushroom industry has also continued to grow (Cao, 2017). According to statistics, in 2022, China exported 1.376×10^5^ tons of shiitake cultivation sticks, and the total annual production of shiitake mushrooms reached 1.296×10^7^ tons, accounting for more than 90% of the global output (Cao et al., 2024). Currently, the domestic shiitake mushroom substitute cultivation has formed the industrial path of industrialized cultivation stick production and ecological fruiting”. By building a standardized service system for strains, formulations, and cultivation materials, enterprises have significantly improved production efficiency and stick quality and lowered the technical barrier for mushroom cultivation compared to the traditional workshop model (Cao et al., 2024). Although the adoption rate of this model in the industry is about 10% as of 2019, it has shown a broad promotion prospect due to its advantages in standardization and scalability (Cao et al., 2024).

With the advancement of shiitake mushroom smart factory construction, related research has mostly focused on environmental control and intelligent harvesting (Wang et al., 2024). However, despite these advances, dynamic monitoring of the cultivation process remains limited. Physiological maturity, a key growth indicator of shiitake cultivation sticks, involves the transformation of mycelium from vegetative to reproductive growth. This process mainly includes three phases: mycelial growth, browning and primordium differentiation (Royse et al., 2017). Among these, mycelial browning is not only an important physiological prerequisite for the formation of the fruiting body but also closely related to yield and quality (Pang et al., 2016). However, currently, the degree of browning mostly relies on manual subjective visual inspection, lacking objective, real-time, and quantitative assessment methods, which makes it difficult to meet the standardized requirements of large-scale factory and commercial production.

To address this, this study proposes a hybrid detection model based on deep learning, aiming to improve the segmentation accuracy and detection efficiency of the browning region on shiitake cultivation sticks. The main research objectives include:

1. To develop a lightweight contour recognition algorithm to extract the contour of shiitake cultivation sticks and effectively eliminate background interference.
2. As the browning region on shiitake cultivation sticks is more difficult to label manually with Labelme, to establish a method based on traditional machine learning to label the browning region of the fungus stick quickly and efficiently by using the ImageJ plug-in module.
3. To establish an algorithm based on the improved U-Net framework to segment the browning region and calculate the percentage of browning to quantitatively assess the degree of browning.

## 2 Related work

In the process of agricultural intelligence, machine vision technologies (Kamilaris and Prenafeta-Boldú, 2018) are increasingly being applied to tasks such as crop maturity and disease detection, gradually reducing the reliance on traditional manual assessment methods. These methods are divided into two main categories: traditional image algorithms and image algorithms based on deep learning.

In the early stages, researchers mostly relied on traditional image algorithms to achieve object detection and classification. Tillett and Batchelor (1991) proposed a method based on height threshold center estimation, low-threshold contour tracking, and region growing for detecting mushroom fruiting bodies. Vizhányó and Felföldi (2000) proposed a method to identify abnormal mushrooms based on color features. Additionally, Yu et al. (2005) proposed a target contour tracking method based on Fourier descriptors of object boundaries. However, these methods are primarily designed for fruiting bodies and are not suitable for accurately quantifying the continuous and gradual browning process of shiitake cultivation sticks. Moreover, they exhibit limited generalizability because they are sensitive to variations in lighting and background, and rely heavily on handcrafted feature extraction (Xiao et al., 2023; Kamilaris and Prenafeta-Boldú, 2018).

In recent years, deep learning technology has gradually emerged in the field of agriculture, in which YOLO series algorithms cover object detection and segmentation tasks, which show great advantages in plant phenotyping, especially in the adaptive detection maturity detection in agricultural products. Chen et al. (2025) extracted the average diameter and length features of asparagus images after ROI segmentation using YOLO-V9. Mandava et al. (2024) constructed a coconut maturity recognition dataset and used YOLO-V5s with YOLO-V4Tiny to achieve classification detection. These models rely on manually labeled closed contour regions and perform well in the segmentation of mushroom stick contour instances.

However, the browning region on the surface of mushroom sticks usually has the problems of fuzzy edges, irregular morphology, and subjective labeling. Inspired by fuzzy boundary model in camouflage target detection (Liu et al., 2023; Lyu et al., 2024), this study adopts the U-Net framework to construct the segmentation model for browning region.

The U-Net architecture proposed by Ronneberger et al. (2015) and others provides an effective solution for medical image segmentation. It is especially advantageous in skin lesion image segmentation. Zafar et al. (2020) used ResNet as a U-Net encoder and proposed an automated method for segmenting lesion boundaries in dermoscopy images. Feature extraction is enhanced by deep learning and residual links of ResNet improving the segmentation accuracy of complex lesions. Cao et al. (2021) proposed a Transformer-based encoder and decoder architecture, Swin-UNet, which incorporates multi-layer spatial contextual semantics into the U-Net framework and is mainly applied in multi-organ segmentation. Zafar et al. (2024) proposed a hybrid architecture combining YOLOv8s and U-Net for fast tumor region identification and fine segmentation of tumor regions. The method achieves initial localization by YOLO and then fine segmentation by U-Net, with a detection accuracy of more than 95%. In addition, Yılmaz et al. (2025) evaluated the robustness of multiple deep learning models under corrosive perturbation conditions and found that the ResNet50-UNet model achieves high Mean Intersection over Union (mIoU) ratios, which can be more than 90%, in several image processing tasks. Zhu et al. (2022) proposed a pixel-level segmentation method using the semantic segmentation network DeepLabv3+, which showed high accuracy under different backbone networks, in which DeepLabv3+ based on RestNe-50 achieved 96.32% segmentation accuracy, realizing stable and effective fabric defect detection. Based on the good performance of the above methods in medical and industrial image processing, shiitake mushroom stick images are difficult to directly migrate to the existing model due to the characteristics of fuzzy browning boundaries, variable morphology, and uneven browning, as well as the lack of objective standards for annotation and the existence of artificial subjectivity. Coupled with the high real-time and deploy ability requirements of factory cultivation, targeted improvement strategies are urgently needed.

## 3 Materials and methods

### 3.1 Data

#### 3.1.1 Data collection

**Figure 1:**
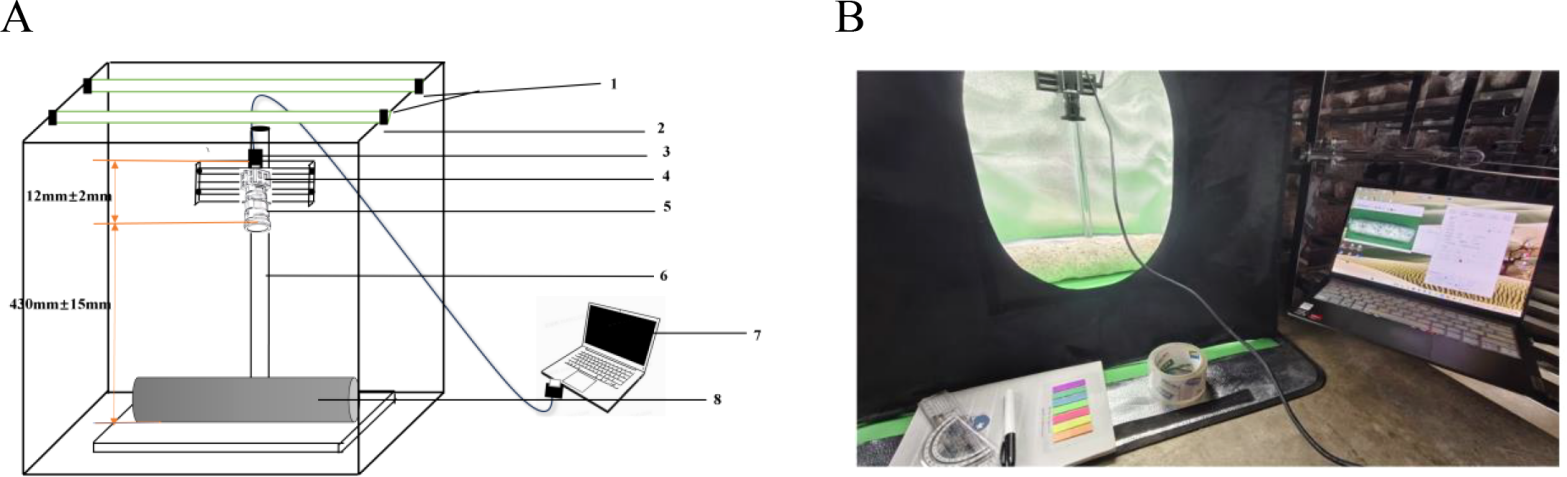
Sketch and physical drawing of the mushroom stick imaging device. **(A)** Device structure diagram: 1.LED light source 2. Rectangular camera box 3.USB hub 4. Camera 5. Lens 6. Fixed bracket 7. Laptop 8. Mushroom Sticks. **(B)** Physical image of the device.

**Figure 2:**
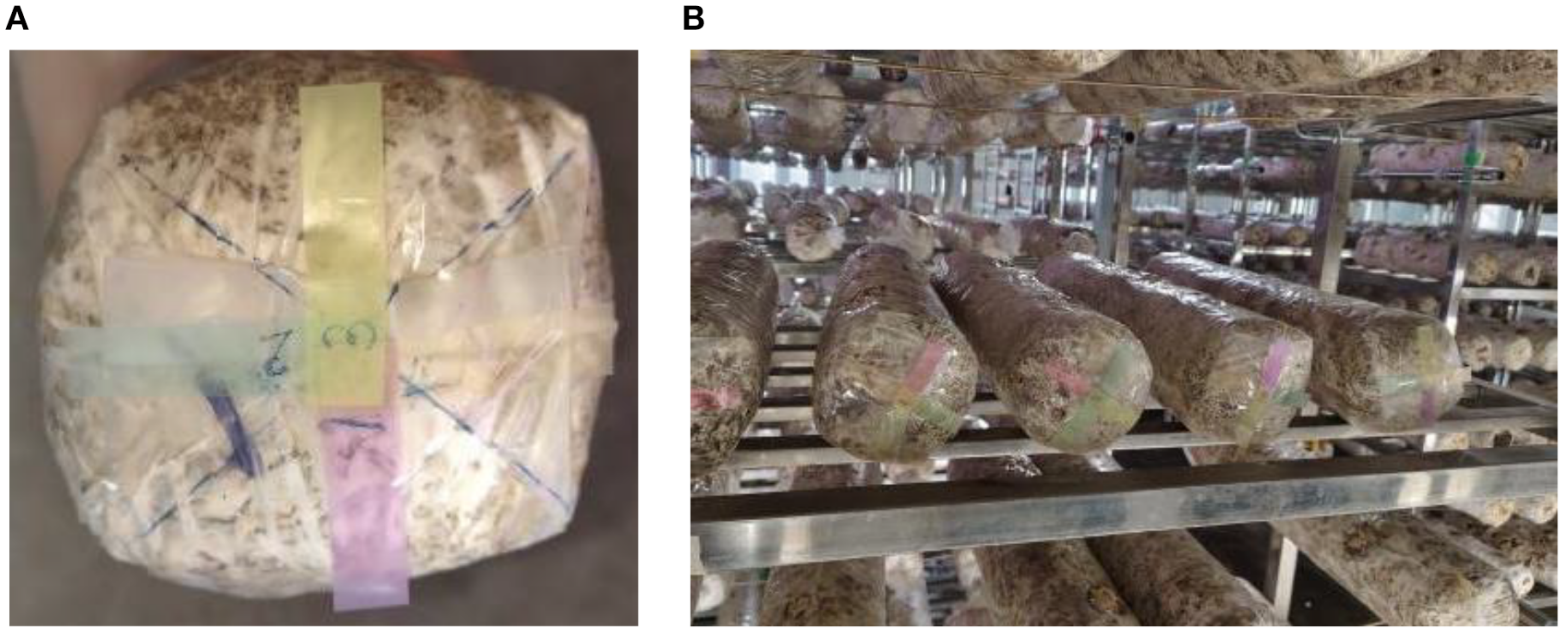
Schematic diagram of the bottom marking of the sticks (divided into four parts). **(A)** Diagram of labeling on the bottom of the stick. **(B)** Example diagram of the sticks after labeling.

**Figure 3:**
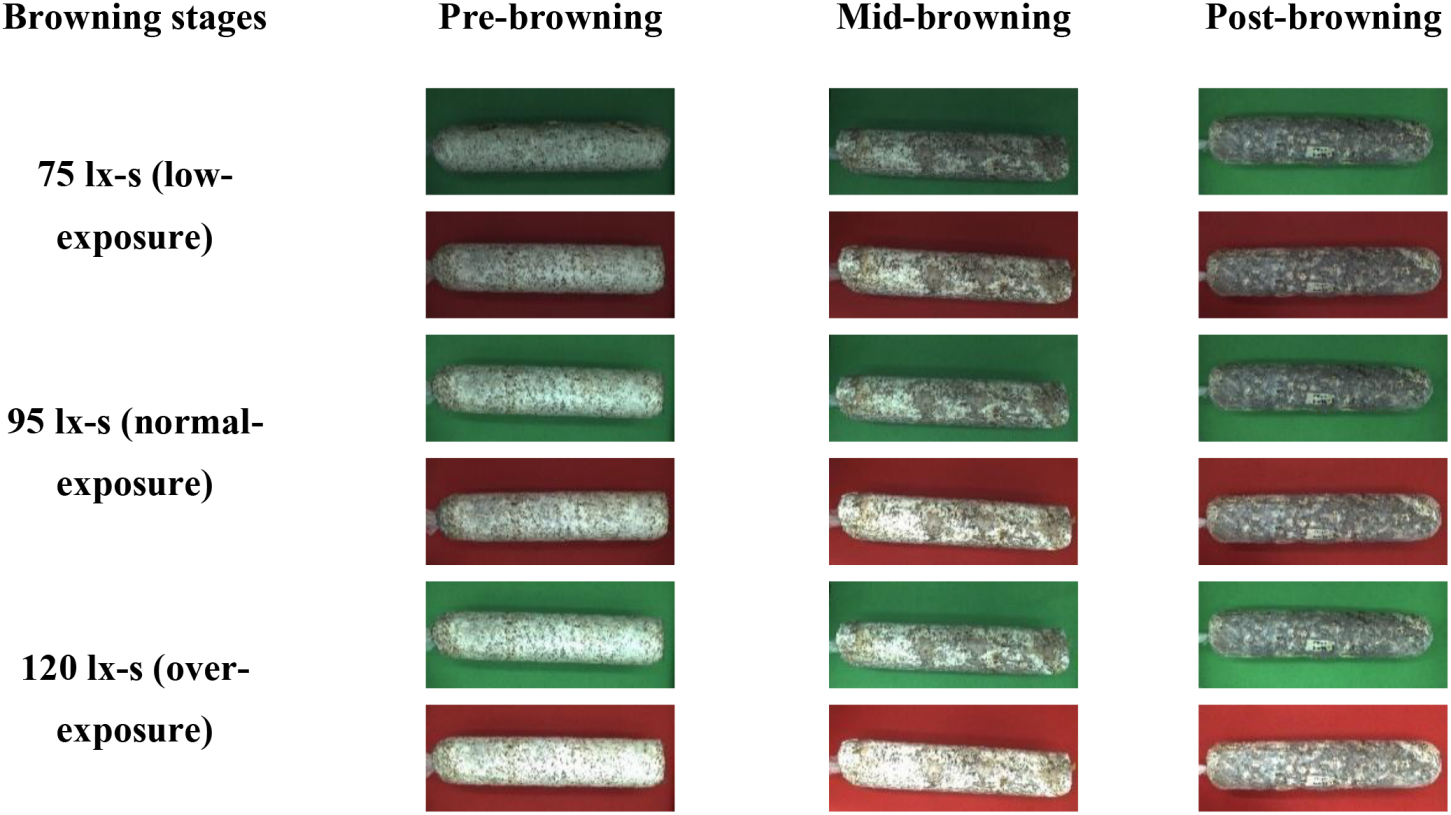
Data acquisition diagram of shiitake cultivation sticks.

The browning image data of shiitake cultivation sticks were collected from March 5 to June 16, 2025, within the edible mushroom experimental station (39.94°N latitude, 116.28°E longitude) of the Beijing Academy of Agriculture and Forestry Sciences (BAAFS). The Jingke No. 1 shiitake mushroom stick variety was selected as the research subject, and the data were acquired with an independently constructed image acquisition device. The device, shown in **Figure 1**, consists of a camera box equipped with a double-LED light board (60cm×60cm×60cm), a MindVision MV-SUA2000C high-definition industrial camera (with an MV-LD-8-25M-A fixed-focus lens featuring low-light sensitivity and a large aperture), and a Lenovo Xiaoxin Air14 laptop. The camera is mounted on a fixed bracket with a constant distance of 43.2cm between the lens and base, connected via USB. During the experiment, based on the approximate tetrahedral structure of the mushroom stick, we divided it into four imaging regions by marking the bottom diagonal and captured images every 90° of rotation (**Figure 2**). The images were stored in.JPEG format with a resolution of 4864 × 2088 pixels. To thoroughly record the browning process, we divided it into three stages: pre-browning, mid-browning, and post-browning (**Figure 3**). By fixing light intensity, setting red and green backgrounds, and adjusting camera exposure parameters (brightness target values of 75 lx·s, 95 lx·s, and 120 lx·s), we created a comprehensive dataset. A total of 1,254 valid images were obtained throughout the longitudinal recording of the browning process. The number of images corresponding to the pre-browning, mid-browning, and post-browning stages is 251, 690, and 313, respectively. This stage-specific distribution accurately reflects the biological progression of browning, thereby ensuring both the diversity and representativeness of the dataset.

#### 3.1.2 Data preprocessing

**Figure 4:**
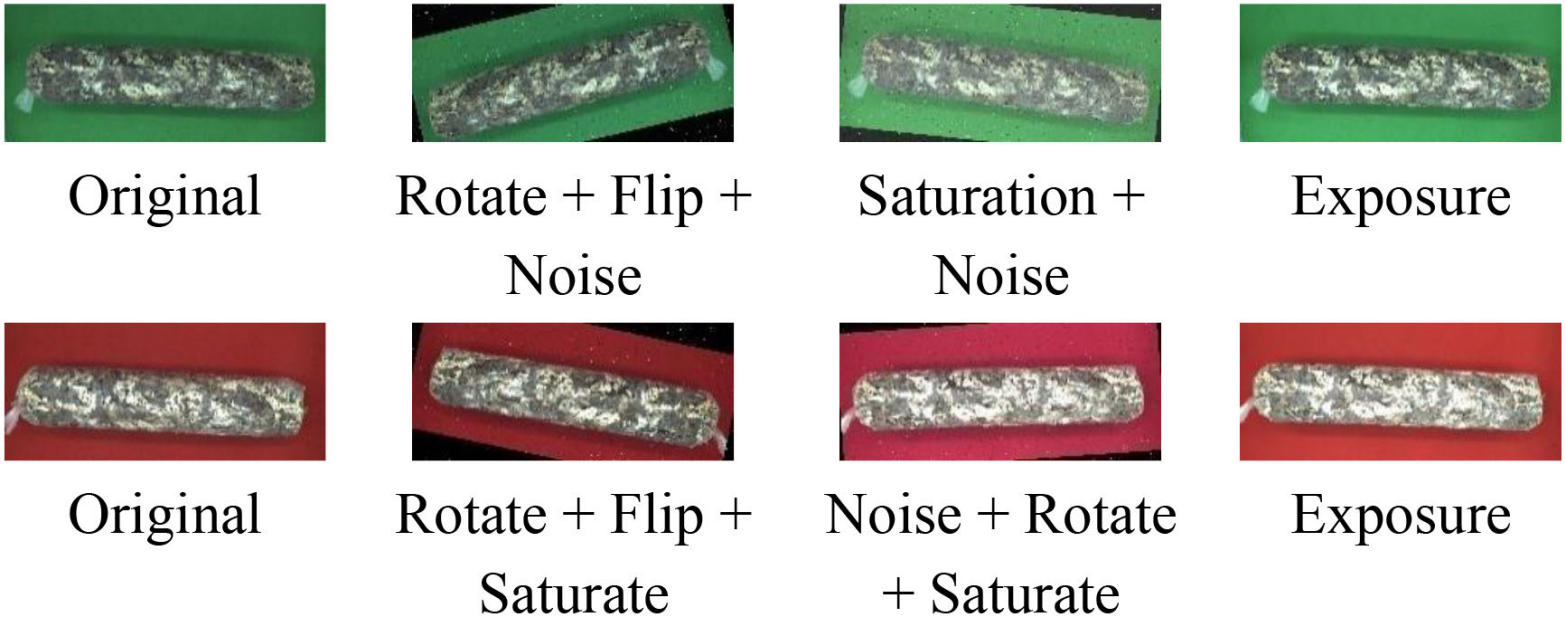
Illustration of data augmentation.

The 1254 original mushroom stick images were labeled with stick contour categories using Labelme v4.5.13. To improve the robustness and generalization ability of the network model, the labeled shiitake stick images were augmented (Javanmardi and Miraei Ashtiani, 2025) by rotating them in multiple angles (± 15°, ± 10°), adjusting brightness contrast (± 3%, ± 5%, ± 7%), adding noise (1%), flipping them horizontally, and adjusting exposure (± 8%) and saturation (± 3%), through random combinations (**Figure 4**).The 1254 images were expanded to 2508 augmented samples, totaling 3762 images. All data were derived from longitudinal monitoring of the entire browning process across 36 independent biological replicates. The samples were randomly divided into training (2910), validation (376), and test (376) sets in an 8:1:1 ratio for model training and validation.

#### 3.1.3 Experimental environment

This experiment uses Windows system, configured with 12th generation Intel Corei9-12900K processor, 64GB RAM, 1TBN VMe SSD, equipped with Nvidia GeForce RTX 3090 graphics card (24GB video memory), and the experimental software environment is set up with Python3.8.16, PyTorch1.13. 1 with CUDA11.7.

### 3.2 Methods

**Figure 5:**
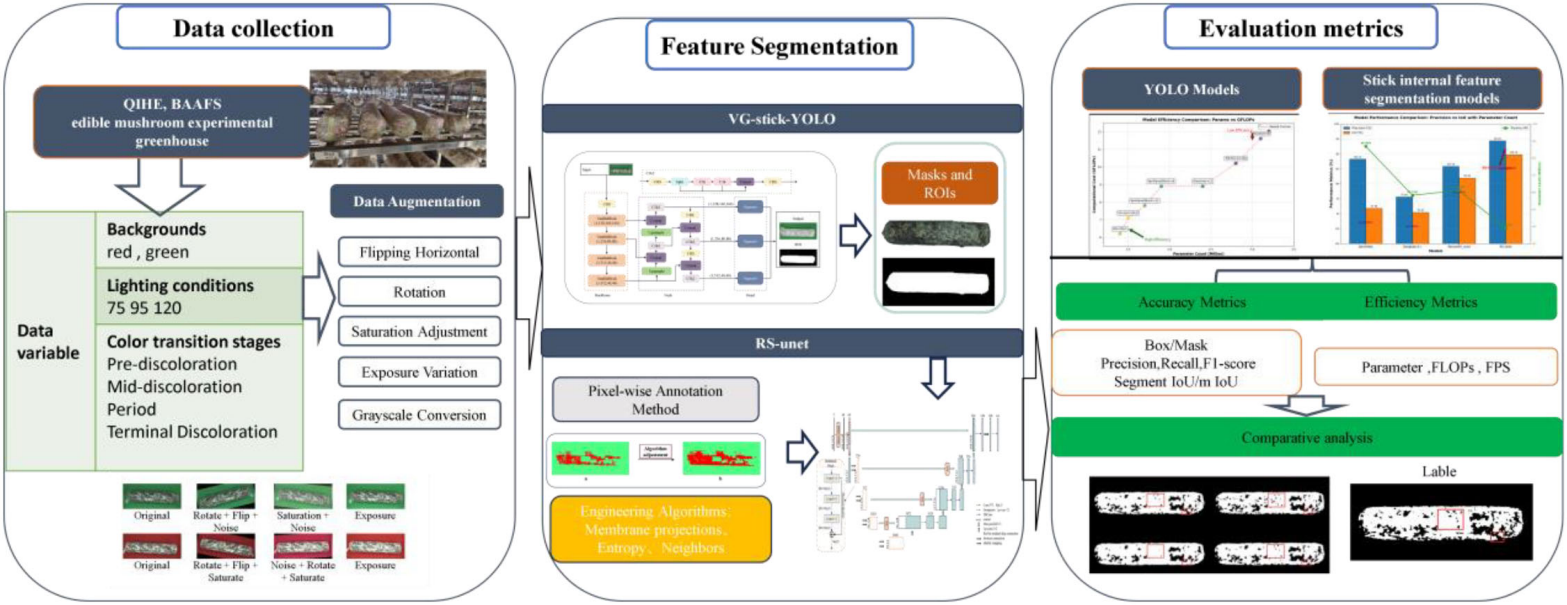
Flowchart of maturity detection experiment for shiitake sticks during browning period.

This study aims to precisely assess the browning maturity of shiitake cultivation sticks in complex surface environments to promote its application in precision cultivation and factory management. As shown in **Figure 5**, the overall experimental process includes three major modules: data collection, feature extraction, and model evaluation. First, RGB images of mushroom sticks during browning stages were collected by a high-resolution camera and data diversity was enhanced by applying data augmentation strategies such as rotation, flipping, and color perturbation. A two-stage deep learning algorithm is used for feature extraction. In the first stage, the VG-Stick-YOLOv11 model is used for detecting the contours of the mushroom stick and generate masks to obtain the regions of interest (ROI). Machine learning algorithms are combined to generate high-quality training labels and complete the dataset of the browning regions. In the second stage, the improved RS-UNet model is trained based on the browning region dataset to perform fine segmentation and extract color and texture features, thereby quantifying the proportion of the browning on the mushroom sticks. Finally, the model performance is comprehensively evaluated using evaluation metrics such as IoU, accuracy, and inference efficiency, along with qualitative visualization analysis. The following sections describe the details of the algorithm and the specific methods used in the above two stages.

#### 3.2.1 VG-stick-YOLOv11

**Figure 6:**
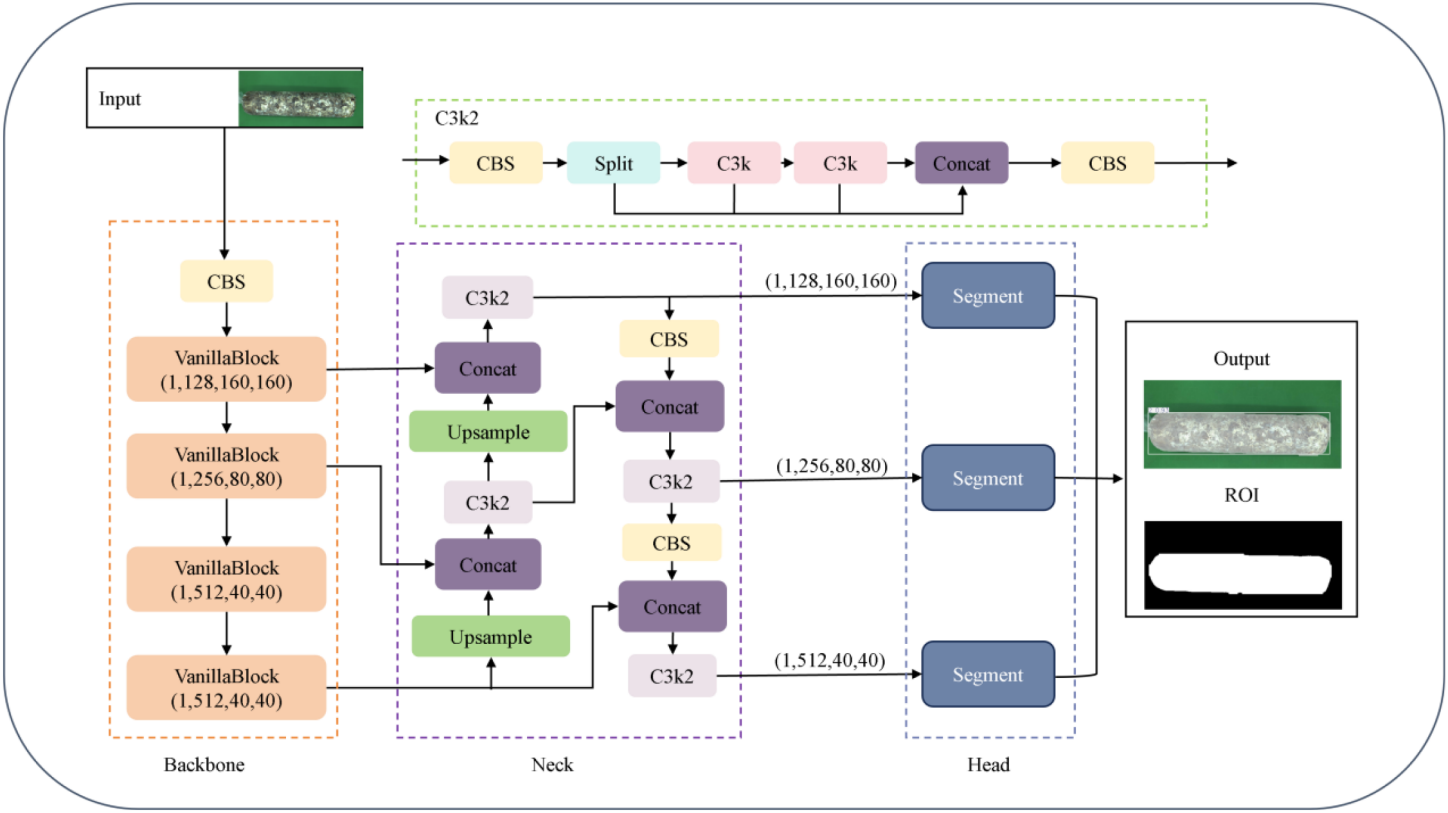
VG-stick-YOLOv11 model structure.

To achieve model lightweight and real-time detection accuracy, this paper adopts the YOLOv11n-seg framework to construct the VG-Stick-YOLOv11 model (**Figure 6**), which is used for the segmentation of the mushroom stick’s contour and the extraction of the ROI.YOLOv11 belongs to a single-stage object detection framework. Its segmentation extension network typically includes three parts: the backbone network for feature extraction, the feature fusion layer (Neck) for multi-scale information fusion, and the detection layer (Head)responsible for object classification, localization and segmentation. Among them, the C3K module integrates depthwise separable convolution (DSC) (Howard et al., 2017) and self-attention mechanism (Cheng et al., 2021). Compared with the C2f module in YOLOv8, it introduces cross-stage connection and lightweight attention design, thus improving the feature extraction effect and reducing the number of model parameters. The head adopts a decoupled design to separate classification and regression tasks, removes the traditional object branch, utilizes the fully convolutional structure to achieve anchor-free target localization and dynamic label assignment. At the same time, an instance segmentation module is integrated to directly predict pixel-level masks based on multi-scale features. Considering that mushroom stick browning detection requires both real-time performance in practical production environments and accurate instance segmentation of stick contours, YOLOv11n-seg provides a suitable balance between accuracy and efficiency, making it an appropriate baseline for constructing the improved VG-Stick-YOLOv11 model.

Based on the lightweight improvement requirements, VG-Stick-YOLOv11 introduces GhostNet convolution (GhostConv) to replace the standard convolution (Han et al., 2020) in the segmentation head of YOLOv11 framework. Compared with Depthwise Separable Convolution (DSC), GhostConv generates richer feature maps to improve segmentation accuracy. By combining standard convolution with linear operation, it improves the feature expression ability while maintaining parameter efficiency, achieving a good balance between lightweight and performance.

**Figure 7:**
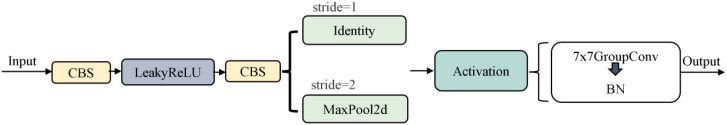
VanillaNetBlock structure.

The VanillaNetBlock module from VanillaNet (Chen et al., 2023) is integrated into the YULOv11 backbone network, replacing the residual connection, attention mechanism and feature pyramid structure in YOLO, realizing lightweight design while maintaining accuracy. In this regard, the dynamic ReLU activation function enables the model to realize structure-aware optimization under hardware-friendly conditions, improving model deployment efficiency. For the SPPF module used for multi-scale semantic fusion and receptive field expansion in YOLO structure, there is a problem of high computational complexity. VanillaNetBlock decouples multi-branch features via grouped convolution and enhances semantic representation with dynamic activation, compensating for the loss of information fusion after removing SPPF. To address the high computational complexity of the SPPF module for multi-scale semantic fusion and receptive field expansion in YOLO structure (**Figure 7**).

#### 3.2.2 ROI-guided semi-automatic annotation of browning regions

To construct high-quality pixel-level annotated labels, this study proposes a semi-automatic annotation method based on the ROI of the stick contours, combining manual interaction with machine learning classification. Browning and non-browning regions are manually selected as training samples to build a binary classification task. Multi-scale texture, edge, and color features are extracted for each pixel, and these features are used to train a pixel-level classification model for accurate segmentation of browning regions.

Specifically, color features are derived from multi-channel information such as RGB and HSV, while texture and edge features are enhanced using a variety of image processing operators. The difference of Gaussian (DoG) is used to enhance edge responses, and is calculated as shown in Equation 1 (Lowe, 2004):

(1) $$DoG(x,\;y)=G_{\left(\sigma 1)(x,\;y\right)}-G_{\left(\sigma 2)(x,\;y\right)}$$

where G_(σ) (x, y) denotes the 2D Gaussian blurring result with a standard deviation of σ, which is used to enhance the contrast of the browning boundary. Membrane Projections simulate the longitudinal texture pattern of the surface tissue, aiding in the identification of striped browning regions on the surface of the sticks.

In addition, the image Entropy measures the local complexity of an image and is defined as shown in Equation 2 (Ronneberger et al., 2015):

(2) $$H(x,y)=-\sum_{i=1}^{N}p_{i}\log_{2}p_{i\;}$$

where $p_{i}$ denotes the ith class gray values probability in the pixel’s neighborhood. High-entropy regions tend to correspond to browning regions with drastic color variations. Neighborhood analysis further captures the structural continuity near the browning edges and improves the model’s ability to identify the boundary regions.

In terms of the classification model, this method uses Random Forest as the pixel-level discriminative model, which inputs the feature vector $x_{i}=[f1,\;f2,\ldots ,fn]$ for each pixel and outputs its corresponding category label $y_{i}\in \{0,1\}$, representing the browning and non-browning regions, respectively. The integrated structure of Random Forest enhances the model’s robustness and generalization ability. This semi-automatic labeling method for browning regions balances efficiency and accuracy, retaining the discriminative ability of manual supervision, while makes full use of the automatic identification advantage of machine learning models.

#### 3.2.3 RS-Unet

**Figure 8:**
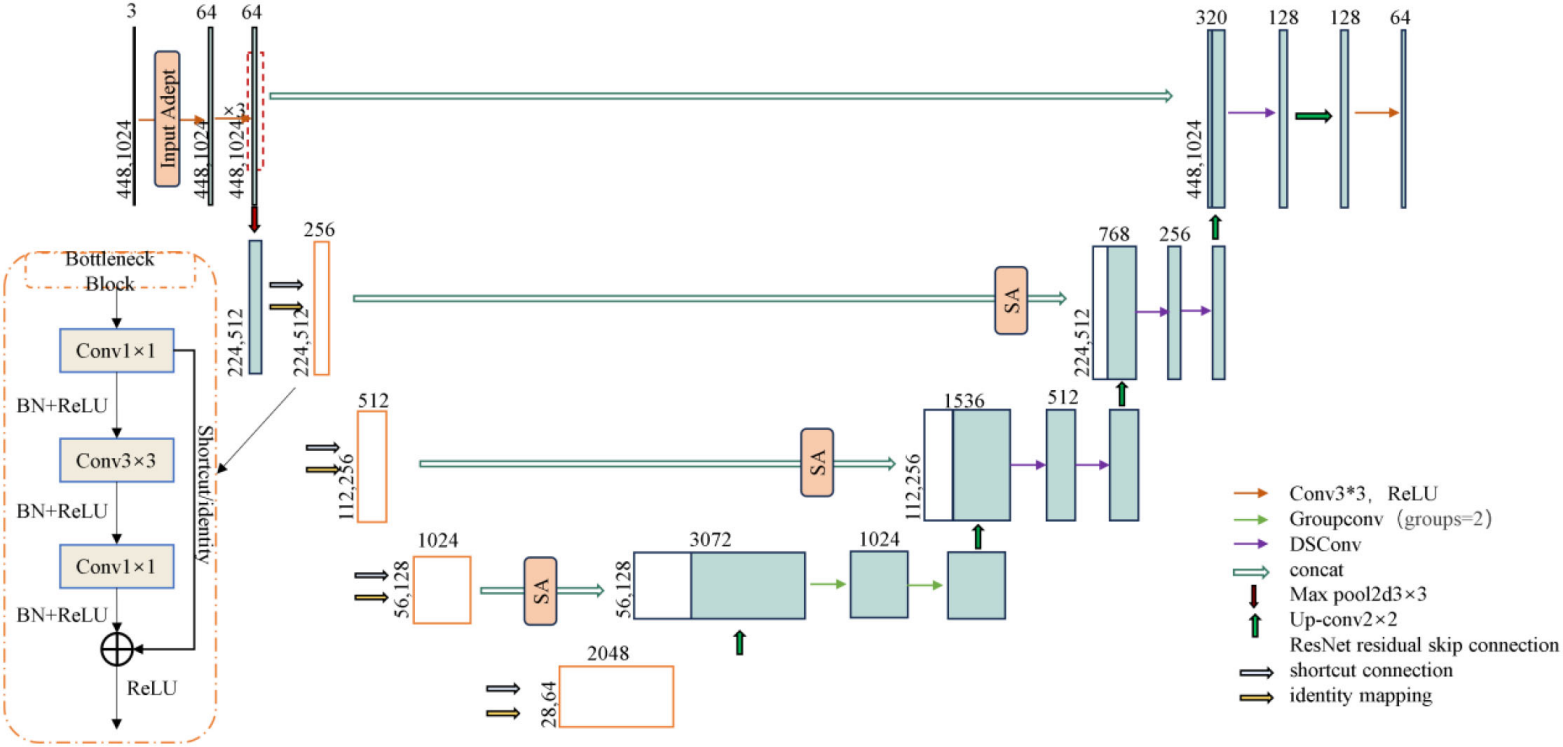
RS-UNet model structure.

Based on the U-Net (Cui et al., 2024) framework, this study constructs a segmentation model for the browning region of shiitake mushroom stick. ResNet50 is used as the encoder, integrating deep residual learning with a U-shaped symmetrical architecture, to effectively improve the feature extraction ability and network expression depth. To address the multi-scale features of mycelial color gradients and texture diffusion in the browning region, a feature enhancement strategy is incorporated into the encoder to improve the network’s perception of browning-related patterns. In the decoder, a hybrid convolution structure—combining grouped convolution and depthwise separable convolution—is designed to achieve efficient channel fusion via pointwise convolution, thereby reducing computational complexity while enhancing feature representation. Moreover, the Spatial Attention (SA) module is introduced at the skip connections to dynamically focus on the mycelial pigment deposition areas, thus enhancing the sensitivity to boundaries and segmentation accuracy. The improved model is named ResNet-Stick-UNet (RS-UNet), and its network structure is shown in **Figure 8**.

**Figure 9:**
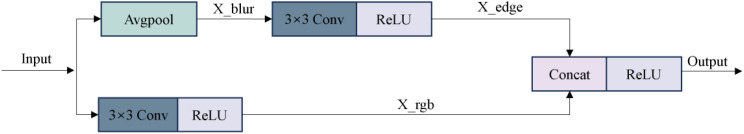
Input adapter structure.

Based on the U-Net framework, this study constructs a segmentation model for the browning region of shiitake mushroom stick. ResNet50 is used as the encoder, integrating deep residual learning with a U-shaped symmetrical architecture, to effectively improve the feature extraction ability and network expression depth. To address the multi-scale features of mycelial color gradients and texture diffusion in the browning region, a feature enhancement strategy is incorporated into the encoder to improve the network’s perception of browning-related patterns. In the decoder, a hybrid convolution structure—combining grouped convolution and depthwise separable convolution—is designed to achieve efficient channel fusion via pointwise convolution, thereby reducing computational complexity while enhancing feature representation. Moreover, the Spatial Attention (SA) module is introduced at the skip connections to dynamically focus on the mycelial pigment deposition areas, thus enhancing the sensitivity to boundaries and segmentation accuracy. The improved model is named ResNet-Stick-UNet (RS-UNet), and its network structure is shown in **Figure 9**.

##### 3.2.3.1 Encoder optimization

Considering the insufficient sensitivity of the traditional ResNet encoder to fine-grained texture changes and edge details in the browning region of mushroom sticks, this paper optimizes the initial module of the encoder. Specifically, the original 7×7 convolutional kernel in ResNet50 is replaced with a triple-layer 3×3 convolutional module to enhance the nonlinear representational capacity and expand the effective receptive field, thereby improving the extraction of complex color changes and texture features in the browning region. In addition, a Input Adapter module is introduced to the input of the network to perform edge-and texture-based preprocessing on the original RGB images. This guides the model to focus on key surface regions of the shiitake cultivation sticks, while suppressing background interference (e.g., plastic bags, labels), thereby improving the subsequent segmentation accuracy. As illustrated in **Figure 9**, this module adopts a dual-branch parallel structure. The lower branch directly processes the original input using 3×3 convolution to preserve color information, and its computational process is as follows (Equation 3):

(3) $$\begin{array}{c} \begin{array}{c} F_{rgb}=ReLU \end{array} \end{array}(W_{rgb}*X+b_{rgb})$$

Specifically, $W_{rgb}\in {\mathbb{R}}^{16\times 3\times 3\times 3}$ denotes the convolution kernel weights, and the output feature map is $F_{rgb}\in {\mathbb{R}}^{16\times H\times W}$. The edge detection branch enhances texture features by calculating the difference between the original image and its 3 × 3 average pooling result. The convolutional kernel weights in this branch are denoting as ${\text{W}}_{\text{edge}}\in {\mathbb{R}}^{16\times 3\times 3\times 3}$, and the output is a texture-enhanced feature map. After concatenating the outputs of the two branches in the channel dimension, the 3×3 fusion convolution is used for feature integration (Equation 4):

(4) $$F_{fuse}=\;ReLU(W_{fuse}\cdot [F_{rgb},\;F_{edge}]+\;b_{fuse})$$

The fusion convolution kernel is denoted as ${\text{W}}_{\text{fuse}}\in {\mathbb{R}}^{64\times 32\times 3\times 3}$, and the output enhanced feature map is ${\text{F}}_{\text{fuse}}\in {\mathbb{R}}^{64\times H\times W}$. All convolutional layers are followed by ReLU activation function to introduce nonlinearity. This structural design enables the network to decouple color and texture information at the initial stage, providing more discriminative feature representation for the segmentation of the browning region.

##### 3.2.3.2 Decoder enhancement

After replacing the encoder with ResNet50, a hybrid convolutional structure is further introduced in the decoder to enhance the representational capacity of low-level semantic features in skip connections and to mitigate potential semantic compression during dimensionality reduction. In the fourth layer of the upsampling module, grouping convolution (Group=2) is applied to the concatenated feature maps with 2048 and 1024channels, enabling separate modeling of color and texture channel groups to improve the recognition of gradient features from light brown to reddish brown.

**Figure 10:**
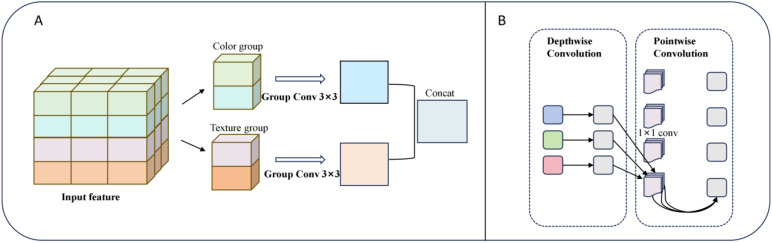
**(A)** Depthwise separable convolution structure. **(B)** Group convolution structure (groups=2).

Specifically, as shown in **Figure 10A**, grouped convolution efficiently extracts distinct features and enhances the model’s sensitivity to subtle browning process. In the middle and low-level feature reconstruction stage, the DSC structure (**Figure 10B**) is introduced. In this structure, depthwise convolution captures the spatial distribution pattern of mycelial pigment secretion, while 1×1 pointwise convolution enables efficient channel-wise reorganization. This design significantly reduces the number of while maintaining robustness to the complex surface texture of shiitake sticks, such as cultivation substrate and plastic bag wrinkles (Xu et al., 2024). Compared with the direct channel compression following skip connections, the hybrid convolutional structure further enhances the model’s discriminative ability and detail retention without compromising computational efficiency.

Furthermore, to enhance model robustness and prevent overfitting, this study introduces a spatial adaptive regularization strategy before the output of the decoder’s final two feature map layers. A 30% spatial Dropout is applied to randomly mask local feature, suppress redundant response, and encourage the network to focus on informative areas. Combined with hybrid convolution, this design proves especially suitable for the mushroom stick dataset, enhancing the model’s segmentation performance while preventing overfitting.

##### 3.2.3.3 Add attention mechanism spatial attention

**Figure 11:**
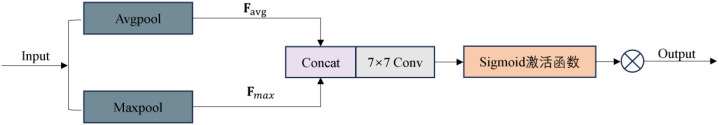
Spatial Attention Structure.

To improve the decoder’s responsiveness to key regions, this study incorporates a SA mechanism into the skip connections during the decoding stage (Fu et al., 2019). As shown in **Figure 11**, the SA module extracts muti-scale spatial information from the browning regions by combining max polling and average pooling, enabling it to captures salient features from different perspectives. Then, the attention weight map is generated to reweight the input features, allowing the model to focus on the browning regions while suppressing background noise (e.g., surface textures of the cultivation sticks). This design enhances the model’s ability to accurately localize and segment the browning region.

This process can be described by the following equation (Woo et al., 2018): Given the input feature map $F\in {\mathbb{R}}^{C\times H\times W}$, max pooling and average pooling are first performed along the channel dimension to generate two spatial attention maps, as defined in Equations 5, 6, respectively:

(5) $$F_{avg}=AvgPool(F)\in {\mathbb{R}}^{1\times H\times W}$$

(6) $$F_{max}=MaxPool(F)\in {\mathbb{R}}^{1\times H\times W}$$

Next, the two spatial attention maps are concatenated in the channel dimension to obtain the fused features, which is formulated in Equation 7:

(7) $$F_{cat}=Concat[F_{avg},F_{\max}]\in {\mathbb{R}}^{2\times H\times W}$$

Then, spatial attention features are extracted through a 7×7 convolutional layer, as shown in Equations 8, 9:

(8) $$M_{spatial}=\sigma (f^{7\times 7}(F_{cat}))\in {\mathbb{R}}^{1\times H\times W}$$

(9) $$F^{\prime }=F⊗M_{spatial}$$

where [ ;]denotes channel-wise concatenation, $f^{7\times 7}$ represents a 7×7 convolutional operation, σ is the Sigmoid activation function, and ⊗ denotes element-wise multiplication. The output is the feature map after fusing spatial attention.

In the task of browning region segmentation, such regions usually exhibit local aggregation (e.g., brown pigment deposition) and distinct color gradient variation. The spatial attention mechanism effectively assists the model in recovering boundary details and regional shapes (Li et al., 2024). It adaptively enhances salient regions such as browning edges while suppressing background noise, thereby improving segmentation accuracy and boundary clarity.

### 3.3 Evaluation metrics

To comprehensively evaluate the performance of the model, this study adopts a range of evaluation metrics covering both model efficiency and prediction accuracy (Everingham et al., 2015). Specifically, the evaluation includes a series of indicators as following.

(1) In the detection task, Box Precision (BP) is defined as follows (Equation 10):

(10) $$\text{Box Precision}=\frac{TP}{TP+FP},$$

where true positives (TP) refer to the number of predicted boxes correctly matched with ground truth boxes when the Intersection over Union (IoU) is ≥ 0.5. False positives (FP) represent the number of incorrectly predicted boxes with IoU < 0.5, and false negatives (FN) indicates the number of ground truth boxes that were not correctly predicted.

(2) In the segmentation task, mask precision (MP) and recall are calculated based on pixel level as follows (Equations 11, 12):

(11) $$\text{Mask Precision}=\frac{\left|P\cap G\right|}{\left|P\right|},$$

(12) $$\text{Mask Recall}=\frac{\left|P\cap G\right|}{\left|G\right|},$$

Where P is the set of pixels predicted by the model as positive class, Gis the set of ground truth pixels labeled as positive class, and P∩G denotes the intersection of the predicted and the real.

(3) The IoU is a generalized spatial overlap metric for detection and segmentation tasks and is calculated according to Equation 13:

(13) $$\text{IoU}=\frac{\left|P\cap G\right|}{\left|P\cup G\right|},$$

For multi-class segmentation tasks, the mean intersection over Union (mIoU) is the arithmetic average IoU of each class, as defined by Equation 14 (Long et al., 2015):

(14) $$\text{mIoU}=\frac{1}{C}\sum_{i=1}^{C}{\text{IoU}}_{i},$$

Where C denotes the total number of categories, and $”IoU{”}_{i}$ is theIntersection over Union of the i-th category.

(4) Model efficiency is measured by the following four metrics: the number of parameters (total trainable parameters, Params), computation cost (floating-point operations per forward pass, expressed in GFLOPs with an input resolution of 640×640), model size (disk space used to store files, in MB) and inference speed (frames per second, FPS, measured on an NVIDIA GeForce RTX 3090).

## 4 Results

### 4.1 Experimental hyperparameter settings

For a clear comparison of model performance on shiitake stick contour and browning region segmenting tasks, identical hyperparameters were set. The details are presented in **Table 1**.

**Table 1:** Hyperparameter settings.

Hyperparameters	Stick contour segment-value	Browning region segment-value
Learning Rate	0.001	0.0001
Image Size	640x640	1024×448
Dropout	0.2	–
Optimizer	AdamW	AdamW
Batch Size	16	4
Epoch	100	100
Weight Decay	0.0005	–

### 4.2 YOLO results for mushroom stick contour

#### 4.2.1 Comparative experiment

To comprehensively evaluate the segmentation performance of the lightweight improved VG-Stick-YOLOv11 model in shiitake sticks contour extraction, as a test set comprising 376 images was constructed. Two representative models with similar underlying architectures—YOLOv11 and YOLOv8—were selected to comparison. And two backbone lightweight architectures, GhostNet and VanillaNet, were selected to reconstruct the YOLO framework. In addition, through the vanillanet backbone architecture, the performance difference between c3k and c2f was compared in the data. The experimental results are shown in **Table 2**.

**Table 2:** Comparative evaluation of lightweighting improvements.

Model	Params (M)	GFLOPs	Model size (MB)	BP (%)	FPS	MP (%)	MIoU (%)	
YOLOv8n-seg	3.2	12.0	6.6	96.9	96.82	96.9	96.11
YOLOv11n-seg	2.8	10.2	5.8	98.3	77.85	98.3	95.56
GhostNet-v8	3.1	11.6	6.4	82.8	97.32	82.8	95.52
GhostNet-v11	2.4	8.9	5.0	96.1	83.43	96.1	95.34
VanillaNetBlock-v8	1.9	8.9	4.1	98.3	100.62	98.3	95.29
VanillaNetBlock-v11	1.7	7.8	3.7	97.6	86.60	97.6	95.78
VDs-YOLOv11	1.4	6.2	2.9	88.4	72.37	88.4	95.28
VG-Stick-YOLOv11	1.5	7.1	3.5	96.0	85.64	96.0	95.80

As shown in **Table 2**, YOLOv11n-seg achieves a 1.4 percentage point improvement in mask precision, reaching 98.3%, compared to YOLOv8n-seg. Meanwhile, it reduces Params by 12.5% and computational cost by 15%. This demonstrates superior detection and segmentation performance on mushroom sticks while maintaining a more compact model size, thereby verifying the architectural advantages of YOLOv11.

In terms of lightweight backbone structure, VanillaNetBlock outperforms GhostNet under both the YOLOv8 and YOLOv11 frameworks. Taking the YOLOv8 structure as an example, VanillaNetBlock improves box precision by 15.5 percentage points while reducing Params by 38.7% and computational cost by 23.3%. These results indicate that VanillaNetBlock achieves a better balance between model compression and performance.

Among the two improved models constructed based on VanillaNetBlock-v11, VG-Stick-YOLOv11achieves a better trade-off between lightweight and accuracy. Compared with VDs-YOLOv11, it improves the box precision by 8.6% and increases inference speed by 18.4%. Although its accuracy is slightly lower than VanillaNetBlock-v11 (−1.6%), it achieves reductions of 11.8% in Params, 9.0% in GFLOPs, and a 23.4% gain in speed. Moreover, VG-Stick-YOLOv11 achieves the highest mIoU of 95.80% among all models, indicating that its architectural optimizations are more effective in preserving segmentation details of stick contours, making it particularly suitable for real-time deployment within shiitake stick production facilities.

#### 4.2.2 Model visualization

**Figure 12:**
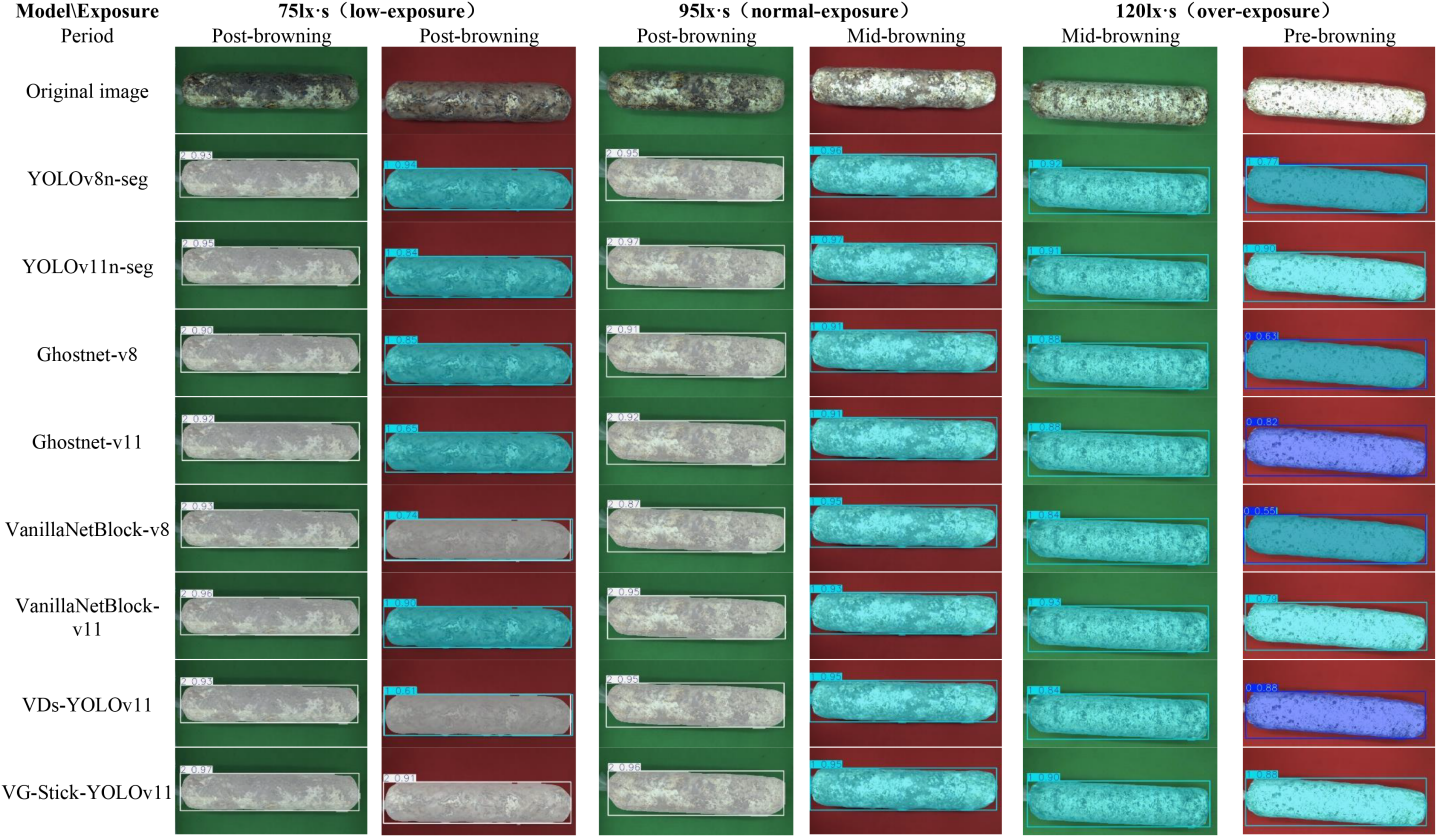
Segmentation results of shiitake stick contours using different model. 0. Pre-browning 1. Mid-browning 2. Post-browning.

To better visualize the segmentation performance of different models under varying exposure settings, background colors, and browning maturity stages, six representative sample images were selected (three with red backgrounds and three with green backgrounds). Object detection and instance segmentation were performed through eight models: YOLOv8n-seg, YOLOv11n-seg, GhostNet-v8, GhostNet-v11, VanillaNetBlock-v8, VanillaNetBlock-11, VDs-YOLOv11, and VG-Stick-YOLOv11. The segmentation results are shown in **Figure 12**.

**Figure 13:**
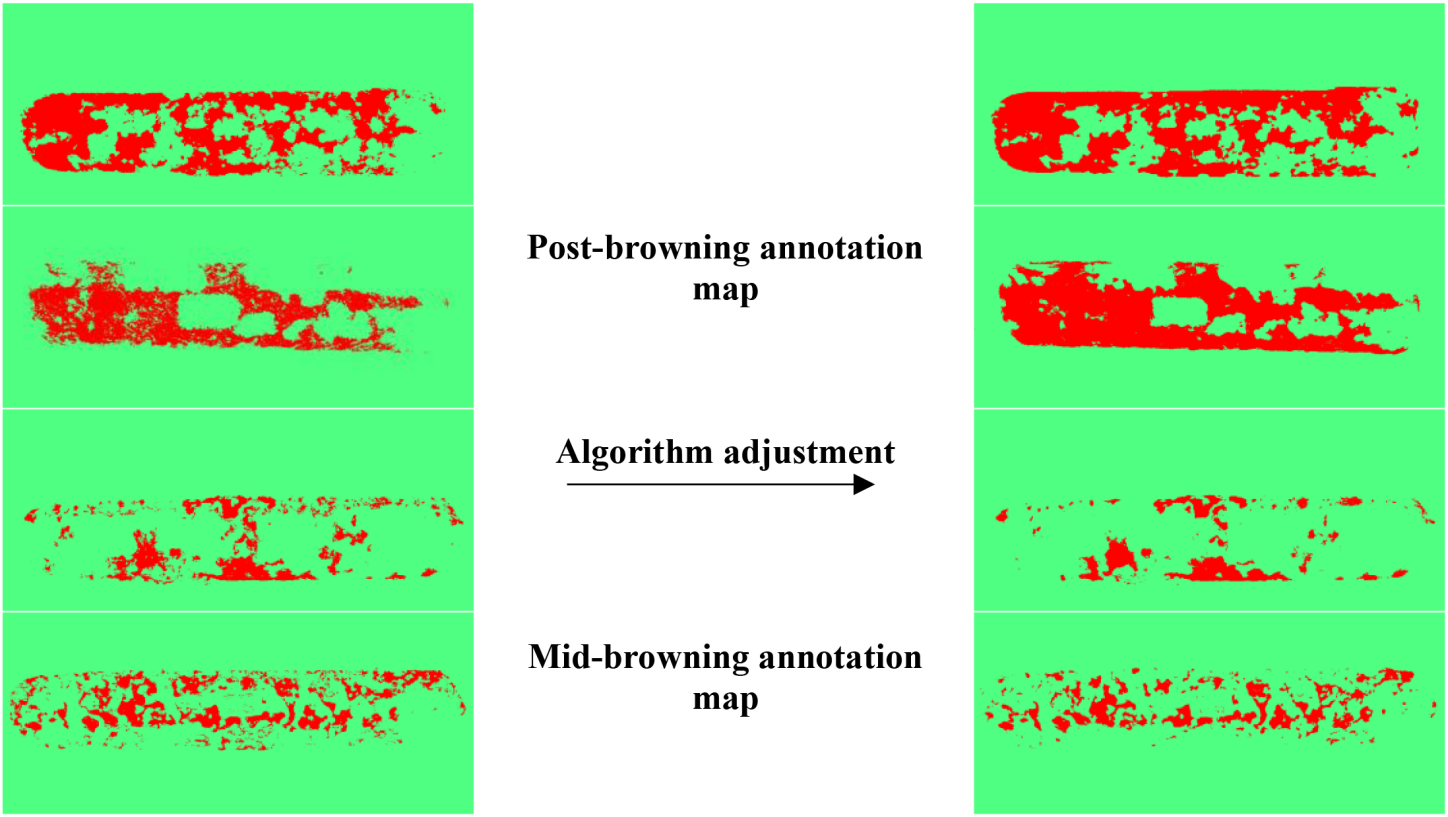
Segmentation comparison of browning areas before and after algorithm tuning.

According to the visual analysis model segment under different background (as shown in **Figure 13**), false segment was observed more frequently under the red background in both low-exposure (75 lx·s) and overexposure (120 lx·s) condition. Compared to the green background, the red background is more lead to overexposure, which mislead to the recognition of samples in low degree browning. Additionally, the reddish-brown appearance of matured samples closely resembles the red background, making accurate segmentation more difficult for most models. However, the VG-Stick-YOLOv11 model can accurately identify in the displayed pictures with a confidence of 0.91. Moreover, under the green background, the segmentation confidence of VG-Stick-YOLOv11 under both low-exposure and normal-exposure (95 lx·s) conditions was significantly higher than under over-exposure. Specifically, for low-exposure samples, VG-Stick-YOLOv11 achieved the highest confidence score of 0.97. Under normal exposure, YOLOv11n-seg produced the highest confidence (0.98), followed closely by VG-Stick-YOLOv11 with a score of 0.96.

### 4.3 Annotation results of shiitake stick browning areas

To verify the effectiveness of semi-automatic labeling based on machine learning, visualization results are shown in **Figure 13**, with four randomly selected. After the introduction of image processing and machine learning algorithms such as DoG, Membrane Projection Transform, and Image Entropy, the generated labeling browning areas results are smoother, with more continuous boundary contours and significantly fewer background mislabels and noise interference compared to those before algorithm adjustment.

### 4.4 Browning area segmentation model for shiitake cultivation sticks

To verify the effectiveness of the improved method, Swin-UNet (Cao et al., 2022), Deeplabv3+, SelfReg-Unet (Zhu et al., 2024) and U-Net are selected as comparison models in this study. Based on this, five sets of ablation experiments were designed around the three improvement points, and all experiments were conducted under the same configuration and training parameters.

#### 4.4.1 Ablation experiment

As shown in **Table 3**, the ablation experimental system evaluates the effect of Encoder Optimization, Decoder Enhancement with SA mechanism on the performance of ResNet50_UNet. In the ResNet50_UNet, the F1 score reaches 87.00%, IoU is 81.91%, and Params is 61.12 M. After the introduction of Encoder Optimization (ResNet50_UNet2), the Precision, Recall, and F1 scores are improved, but the IoU slightly decreases to 80.73%.

**Table 3:** Ablation experiment comparison results.

Experimental group	Encoder optimization	Decoder enhancement	SA	Precision/(%)	Recall/(%)	F1 score/(%)	IoU/(%)	Params
ResNet50-Unet				85.78	88.36	87.0	81.91	61.115329×10^6^
ResNet50-Unet 2	✓			87.51	91.24	89.56	80.73	61.153953×10^6^
ResNet50-Unet 3		✓		93.07	91.03	92.05	86.10	38.831393×10^6^
ResNet50-Unet 4		✓	✓	92.33	93.41	92.86	86.12	38.885349×10^6^
RS-UNet	✓	✓	✓	**94.35**	**92.48**	**93.37**	**88.56**	**38.923973×10^6^**

Bold values indicate the best results across all models.

Based on ResNet50_UNet, decoder enhancement (e.g., hybrid convolution, regularization) significantly improves the model performance and reduces the number of parameters by 36.3%, which achieves a good balance between browning feature expression and computational efficiency. The ResNet50_UNet 4 model, after fusing decoder enhancement with SA module, under original encoder improves Recall, F1 score and IoU compared with ResNet50_UNet3. This suggests that the SA mechanism enhances the ability to capture boundary features by weighting different regions in the feature map, particularly improving the recognition of fuzzy or unevenly browning stick boundaries.

Finally, experimental group RS-UNet combines encoder optimization, decoder enhancement and SA module to further improve the precision, F1 score and IoU. The introduction of SA mechanism effectively compensates for the loss of spatial information caused by the change of feature map size, thereby optimizing the segmentation effect. Compared with the ResNet50_UNet model, the precision, recall, F1 score and IoU of experimental group 5 are improved by 10.0%, 4.66%, 7.32% and 8.12%, respectively, while the parameter volume is reduced by 36.3% to 38.92M.

Overall, the three improvement strategies achieve good synergistic effect in the RS-UNet model, alleviate the conflict between feature extraction, boundary identification and computational cost. This result validates the efficiency and practicality of the improved model under fuzzy boundary conditions.

#### 4.4.2 Quantitative performance comparison of segmentation models

**Table 4** presents the performance comparison between the proposed RS-UNet and several mainstream segmentation models in the task of segmenting the browning region of shiitake cultivation sticks. Based on U-Net achieves, RS-UNet achieves a better balance between accuracy and model complexity, with the F1 score of 93.37% and the an IoU of 88.56%, both of which surpass the other models’ performance. Moreover, RS-UNet maintains a lightweight structure with only 38.92M parameters, presenting a significant advantage over Swin-UNet and DeepLabv3+.

**Table 4:** Comparison of model performances.

Model	Precision/(%)	Recall/(%)	F1 score/(%)	IoU/(%)	Params
Deeplabv3+	75.60	76.57	76.0	70.45	58.234012×10^6^
U-Net	82.57	83.76	83.16	80.89	24.763905×10^6^
Swin-UNet	88.23	78.73	82.89	71.78	86.855739×10^6^
SelfReg-Unet	91,50	90.11	90.79	84.52	31.04150×10^6^
RS-UNet	**94.35**	**92.48**	**​​93.37**	**88.56**	**38.923973×10^6^**

Bold values indicate the best results across all models.

For comparison, **Table 4** also includes the results of several widely semantic segmentation models. Although Swin-UNet achieves a relatively high precision (88.23%), its recall is relatively low (78.73%), indicating that it has leakage and fails to comprehensively cover the browning region; DeepLabv3+ has overall weaker performance, with an IoU of only 70.45%.U-Net, as the baseline model, performs robust performance in the single-class, pixel-level segmentation task, achieving an IoU of 80.89% and Params of 24.76M, thereby achieves a certain balance between accuracy and model size. In addition, SelfReg-Unet achieved F1 score and IoU of 90.79% and 84.52%, respectively, showing strong performance, but its overall performance is still inferior to that of RS-UNet.

Based on this, RS-UNet significantly improves the performance of browning region segmentation while maintaining a reasonable model size, confirming the efficacy of the proposed enhancement strategies.

#### 4.4.3 Visual analysis of boundary segmentation for browning regions

**Figure 14:**
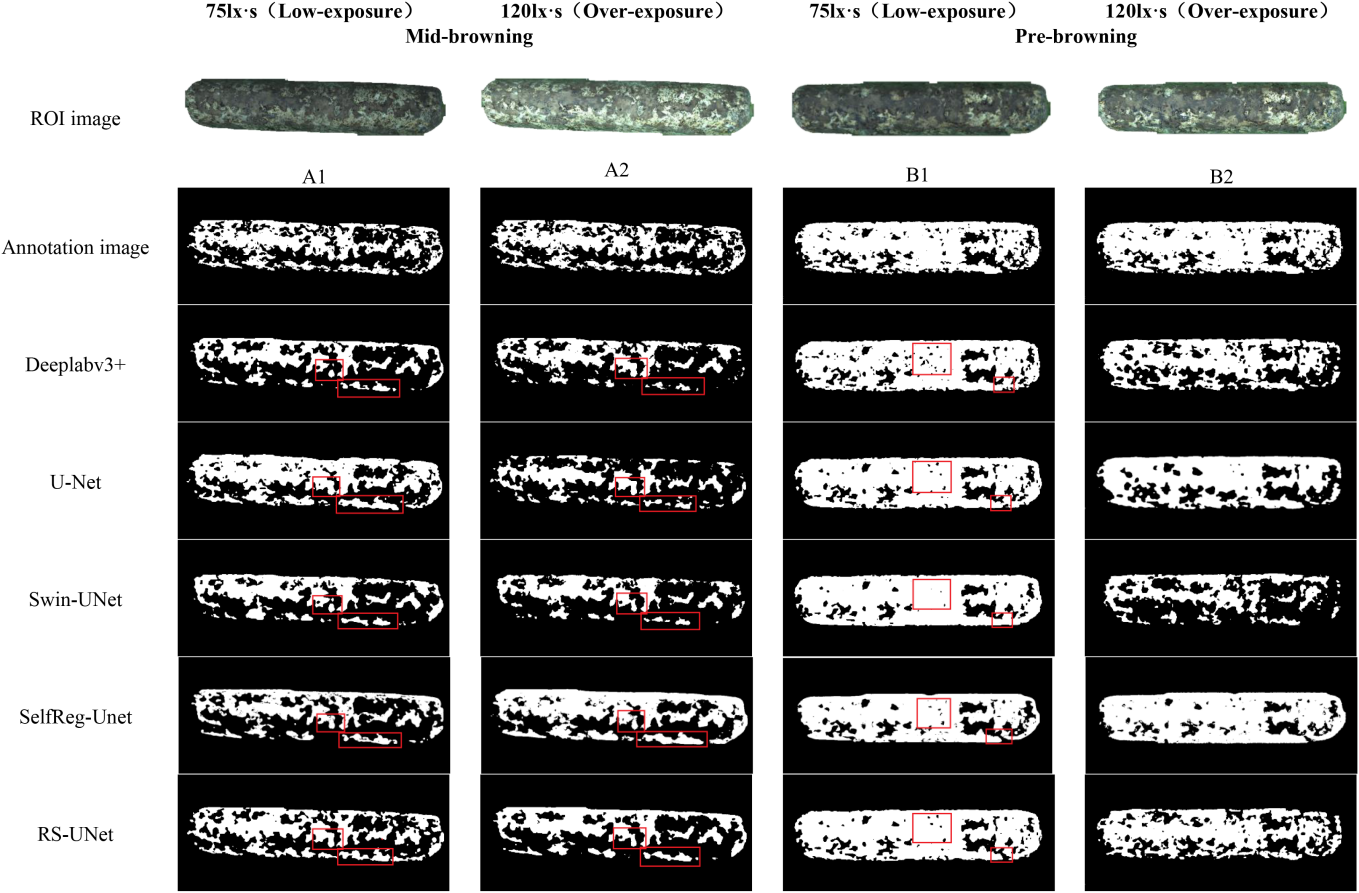
Segmentation effects of browning regions in mushroom sticks under different models.

To verify the effectiveness of the improved RS-UNet network in extracting the boundary of browning regions in mushroom stick images, a comparative analysis was conducted with other segmentation models. As illustrated in **Figure 14**, most models have the problem of incomplete identification of browning regions, and some even incorrectly segment the surface regions of sticks.

**Figure 15:**
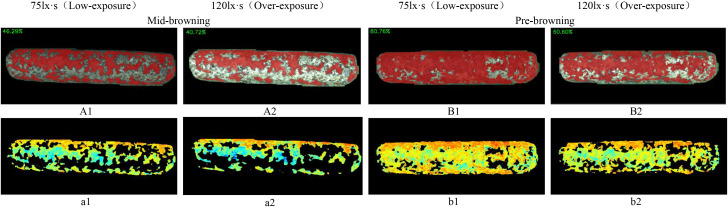
Browning region ratio and pigmentation intensity heat-maps of shiitake cultivation sticks. (**A1, A2, B1, B2**) represent the browning ratio maps. (**a1, a2, b1, b2**) represent the corresponding pigmentation intensity heat-maps.

Meanwhile, this results also validate the effectiveness of pre-segmented stick contours in suppressing background interference. By comparing the segmentation effect under red and green backgrounds (as shown in **Figure 12**), the green background was finally selected for the browning region segmentation. Further analysis of the green background images under varying exposure levels, revealed that under low exposure conditions (**Figures 15A1, 15B1**), all models generally achieved better recognition of the browning regions.

Under overexposure conditions (**Figures 15A1, 15B2**), the color features of the sticks post-browning period and the mid-browning period tended to be similar, resulting in generally incomplete segmentation by the models. Notably, Swin-UNet and Deeplabv3+ performed poor segmentation completeness, althoughDeeplabv3+ showed relatively higher accuracy in identifying hollow areas in the browning region. In comparison, SelfReg-UNet achieved overall good segmentation performance; however, it exhibited partial omission errors under low-exposure conditions.

In contrast, under low-exposure conditions, the proportion of browning regions segmented by each model increased significantly, which was more closely with the labeled images. The RS-UNet model, in particular, exhibited more accurate and well-defined edge segmentation. In addition, compared with overexposure conditions, low-exposure effectively enhances the recognition of the light-colored regions during the mid-browning stage, thereby reducing missed segmentation caused by the weakening of color features under overexposure.

### 4.5 Analysis of browning using hybrid detection

**Figure 15** illustrates the detection results of browning region proportions on shiitake cultivation sticks. The selected images continuous growing process captured from a fixed angle of the same stick, based on RS-UNet visualization results, to display the dynamic transition from the mid-browning stage to the post-browning stage, along with distribution of pigment depth. The results showed that the browning region accounted for 46.29% in the mid-browning sample A1, which increased to 80.76% in the post-browning stage after 14 days of cultivation. Meanwhile, under the overexposure conditions, the proportion of browning (**Figures 15A2, B2**) was significantly lower than that under low-exposure conditions. Furthermore, a comparison of the heatmap a1 (corresponding to A1) and b1 (corresponding to B1) presents a marked expansion of red and yellow regions (representing darker pigmentation), accompanied by a reduction in green regions (lighter pigmentation). This pattern suggests an overall increase in surface pigment deposition and a continuous progression of browning maturity. However, under overexposure (a2, b2), the pigment intensity appeared noticeably lighter.

## 5 Discussion

### 5.1 Experimental evaluation of the two-stage segmentation framework

In response to the questions of detecting the browning ratio in mushroom sticks during factory-scale shiitake mushroom cultivation, this study proposes a two-stage deep learning framework. Through analysis the image characteristics during cultivation of mushroom stick, we find several factors that affect segmentation accuracy. These include the complex visual background—comprising mycelium, culture substrate, and the “yellow water” phenomenon—as well as interference caused by surface bags, particularly in areas with knotting and accumulated folds. Based on these findings, we establish a systematic data processing pipeline and technical optimization scheme, to improve model robustness and segmentation performance.

During the data acquisition phase, we controlled exposure level, background color, and the timing of the browning stage. Experimental results showed that using a green background under low-exposure conditions effectively improved segmentation robustness. In contrast, the red background led to a notable increase in false detection rates, likely due to the high similarity in feature space with the later reddish-brown surface mycelium, this finding consistent with the results reported by Kamilaris and Prenafeta-Boldú (2018) and provides important data collection specifications for subsequent research.

Contour segmentation of mushroom sticks serves a dual purpose in this study. First, by accurately extracting the main region of the stick, it provides data basis for calculating the proportion of the browning region. Second, it effectively eliminates background interference. This step is particularly critical, as stacked plastic bags often visual similarity to the light browning region. Then the plastic film covering the surface, directly labeling the original images can easily result in mislabeling the browning region. Meanwhile, aiming to the demand for lightweight deployment in practical factory environments (Yang et al., 2020), the contour segmentation model was specifically optimized to improve performance maintaining accuracy. Experimental results show that VG-Sticks-YOLOv11 improves 9.9% in FPS over the YOLOv11n-seg baseline, along with a 0.25% percentage point increase in mIoU.

For browning region segmentation, an improved RS-UNet framework was designed by integrating three improvement modules. The hybrid convolution Params is 38.92M, while improving all performance metrics, show its dual advantages in lightweight and feature detection (Xu et al., 2024). Notably, the introduction of regularization strategies could avoid potential over-fitting issues commonly associated with lightweight design (Yang et al., 2020). Compared with U-Net’s symmetric structure, RS-UNet enriches representation; unlike SwinUNet, it avoids the high cost of Transformer modeling while capturing local texture variations; and relative to DeepLabv3+, its spatial attention provides finer localization of pigment spread. This balance between efficiency and accuracy highlights the superiority of the proposed model.

### 5.2 Future work

It should be noted that the data collection in this study has certain limitations. The current dataset includes only single-surface images of mushroom sticks, while in actual production, the stick has three-dimensional structure and the browning process may occur unevenly distribution on all surfaces. So, this single-view acquisition method may limit the comprehensive assessment of the overall browning status, and potentially miss key features present on other surfaces. In addition, additional optimization of the multi-angle imaging system will be required when applied to actual production environment.

Future research will focus on improving data acquisition methods in several aspects. These include the development of a multi-view simultaneous imaging system to capture complete 360° surface information of shiitake cultivation sticks, and the development of a 3D reconstruction algorithm (Wu et al., 2025) to enable more comprehensive assessment of browning status. In addition, imaging setup in the cultivation environment will be further optimized to ensure the reliability of the inspection system in industrial settings. These improvements will help to enhance the system’s accuracy and practicality, thereby better meeting the demands of industrial-scale shiitake mushroom cultivation.

## 6 Conclusion

Under the industrial-scale stick-making mode, the cultivation process of shiitake cultivation sticks is suitable for automated detection based on deep learning, especially in the monitoring of maturity changes during browning phase. Although this research direction holds high application value, relevant research remains scarce. The complex surface structure of mushroom sticks poses greater challenges for segmentation tasks, while lightweight deployment for practical applications is also required. Thus, this study proposes a two-stage collaborative framework that integrates the lightweight VG-Stick-YOLOv11 for contour extraction and the improved RS-UNet for browning region segmentation, we achieve both high efficiency and accuracy. The framework not only improves segmentation robustness under complex visual backgrounds but also provides reliable quantitative indicators of browning ratios, which are essential for maturity assessment and production management.

Furthermore, the adoption of a machine-learning-assisted annotation strategy accelerates dataset construction and enhances the precision of ground-truth labeling, thereby supporting future research in this field. While current work is limited to single-view imaging, the findings demonstrate strong potential for real-world deployment in automated monitoring systems for shiitake cultivation. Future extensions toward multi-view imaging and 3D reconstruction will further enhance comprehensiveness and robustness. This study provides a feasible pathway for the intelligent and standardized detection of browning stages in shiitake cultivation sticks. Since the commercial value of the sticks largely depends on their degree of browning, the proposed method holds promise for offering valuable technical support in quality evaluation and yield improvement under industrial cultivation environments according to Equation 13.

## Data Availability

The raw data supporting the conclusions of this article will be made available by the authors, without undue reservation.
